# Preparation and Characterization of Quaternized Chitosan Derivatives and Assessment of Their Antioxidant Activity

**DOI:** 10.3390/molecules23030516

**Published:** 2018-02-26

**Authors:** Fang Luan, Lijie Wei, Jingjing Zhang, Wenqiang Tan, Yuan Chen, Fang Dong, Qing Li, Zhanyong Guo

**Affiliations:** 1 Key Laboratory of Coastal Biology and Bioresource Utilization, Yantai Institute of Coastal Zone Research, Chinese Academy of Sciences, Yantai 264003, Shangdong, China; fluan@yic.ac.cn (F.L.); ljwei@yic.ac.cn (L.W.); jingjingzhang@yic.ac.cn (J.Z.); wqtan@yic.ac.cn (W.T.); ychen@yic.ac.cn (Y.C.); fdong@yic.ac.cn (F.D.); qli@yic.ac.cn (Q.L.); 2 University of Chinese Academy of Sciences, Beijing 100049, China

**Keywords:** cationic chitosan derivatives, quaternized chitosan, double quaternized chitosan, antioxidant ability

## Abstract

Chitosan (CS) is an abundant and renewable polysaccharide that is reported to exhibit a great variety of beneficial properties. However, the poor solubility of chitosan in water limits its applications. In this paper, we successfully synthesized single *N*-quaternized (QCS) and double *N*-diquaternized (DQCS) chitosan derivatives, and the resulting quaternized materials were water-soluble. The degree of quaternization (DQ) of QCS and DQCS was 0.8 and 1.3, respectively. These derivatives were characterized by FTIR, ^1^H NMR, ^13^C NMR, TGA, and SEM. Moreover, the antioxidant activity of the chitosan was evaluated by free radical scavenging ability (against DPPH-radical, hydroxyl-radical, and superoxide-radical) and ferric reducing power. Our results suggested that the antioxidant abilities were in the order of DQCS > QCS > CS, which was consistent with the number of quaternized groups. These data demonstrate that the number of quaternized groups of chitosan derivatives contributes to their antioxidant activity. Therefore, DQCS, with a higher number of quaternized groups and higher positive charge density, is endowed with high antioxidant activity, and can be used as a candidate material in food and pharmaceutical industries.

## 1. Introduction

Chitin is the second most abundant polysaccharide on earth after cellulose. Chitosan (CS) is produced by the alkaline deacetylation of chitin. CS is reported to exhibit a great variety of beneficial properties, including wound healing, antimicrobial activity, low toxicity, excellent biocompatibility, and antioxidant properties [1,2,3]. Despite its unique antimicrobial properties, the poor solubility of chitosan in water limits its applications. To overcome this problem, a quaternary ammonium moiety was introduced into the chitosan backbone. Quaternized chitosan has two major advantages over the parent chitosan: water-solubility and permanent positive charge. The simplest form of quaternized chitosan, *N*,*N*,*N*-Trimethyl chitosan (TMC), was first reported by Muzzarelliand Tanfani [4], and has wide applications (such as in antimicrobial fields, drug delivery systems, and food applications) due to its better solubility in water compared to CS [5,6]. Usually, there are two synthetic schemes to prepare TMC. One is synthesized with iodomethane and sodium iodide in strong alkaline conditions, using *N*-methyl-2-pyrrolidone as a solvent [7,8]. The other is synthesized with formaldehyde to generate a Schiff-base, followed by a reduction with sodium borohydride and then the addition of methyl halide [9].

Quaternized chitosan derivatives can have excellent antibacterial properties. Amino polysaccharide quaternary ammonium salt has been investigated for applications such as drug carriers (osthol and taxol) and sustained-release drug delivery (amoxicillin, meloxicam, and aspirin) in the field of medicine [10,11,12]. Subsequently, some researchers demonstrated that the antioxidant activity of chitosan and its derivatives should be related to the charge density of the cation [13,14,15]. Amino polysaccharide quaternary ammonium salt is an excellent antibacterial product, which has been investigated for applications such as skin induction, skin douche, nasal lavage fluid, oral rinse fluid, and as a bactericide [16,17,18,19]. Compared to single quaternary ammonium salts derived from chitosan, chitosan with double quaternization may have the potential for wider use in food and medicine fields. Thus, more attention has been attracted to chitosan with double quaternization. To our knowledge, there are few papers about the antioxidant properties of double quaternized chitosan derivatives. Furthermore, the quaternization is based on the nucleophilic substitution of the primary amino group on chitosan. Thus, if we can obtain 6-amino-6-deoxy chitosan and then replace the amino groups in the C-2 and C-6 position, this may result in two sites for quaternization and perhaps higher biological activity.

In the present study, 6-amino-6-deoxy chitosan was prepared in five steps, namely the phthaloylation, bromination, azidation, reduction, and deprotection of the phthaloyl group. We then synthesized the water-soluble chitosan derivatives *N*-quaternized chitosan (QCS) (*N*,*N*,*N*-Trimethyl anmmonium Chitosan Iodide) and *N*-diquaternized chitosan (DQCS) bearing double functional groups. The resulting quaternized materials were water-soluble and had a high degree of quaternization (DQ). Characterization of the prepared samples was conducted using different analyses: FTIR, ^1^H NMR, ^13^C NMR, TGA, and SEM. In our report, antioxidant activity was examined by two different mechanisms—free radical scavenging ability (against DPPH-radical hydroxyl-radical and superoxide-radical), and ferric reducing antioxidant power.

## 2. Results

### 2.1. Chitosan Derivatives Preparation and Characterization

#### 2.1.1. Synthesis of Chitosan Derivatives

**Scheme 1:**
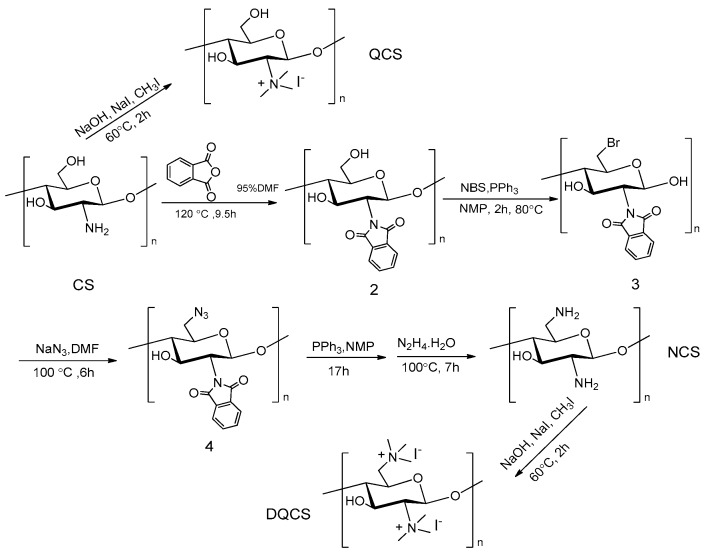
Synthetic route for the preparation of QCS and DQCS.

The reaction shown in Scheme 1 was carried out according to the literature with some modification [20]. The reaction was performed with a single treatment of iodomethane, from which we obtained the derivative as a mixture of *N*-methylated chitosan, *N*,*N*-dimethylated chitosan, and TMC. In order to obtain TMC with high DQ, we repeated the reaction twice with the same quantity of iodomethane.

#### 2.1.2. FTIR Analysis

**Figure 1:**
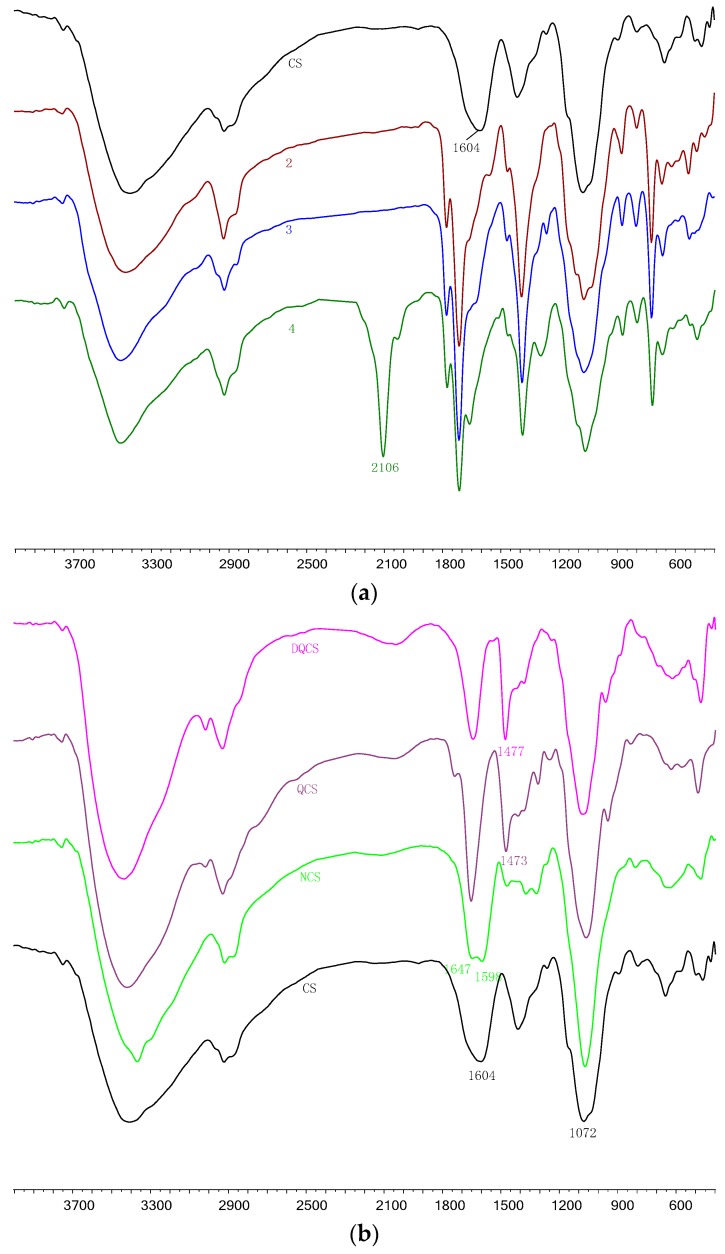
(**a**) FTIR spectra of the chitosan (CS), phthaloylation of chitosan (2), 6-bromo-6-deoxy-*N*-phthaloyl-chitosan (3), 6-azido-6-deoxy-*N*-phthaloyl-chitosan (4). (**b**) IR spectra of the chitosan (CS), 6-amino-6-deoxychitosan (NCS), *N*-quaternized chitosan (QCS), and *N*-diquaternized chitosan (DQCS).

FTIR spectroscopy has been demonstrated to be a powerful tool for the study of the physicochemical properties of polysaccharide. The syntheses were confirmed by the FTIR spectra shown in Figure 1.

For chitosan, the absorption at around 3400 cm^−1^ was assigned to the stretching vibration of –OH and –NH_2_. The band at around 1600 cm^−1^ was assigned to the bending vibration of –NH_2_. The twin strong absorptions at around 1080 and 1030 cm^−1^ were related to the C–O stretching vibration of the second hydroxyl group and primary hydroxyl group, respectively [21]. The spectra of *N*-phthaloyl-chitosan showed two strong characteristic absorptions at 1774 cm^−1^ and 1709 cm^−1^, which were assigned to the stretching vibration of C=O. The peak at 721 cm^−1^ was due to the bending vibration of C–H in the aromatic ring. Although there was no obvious peak at around 530 cm^−1^ in the spectra of compound **3**, the C–O stretching band at 1030 cm^−1^ corresponding to the primary hydroxyl group disappeared, which verified the bromination of the C_6_–OH. For compound **4**, a new peak at around 2106 cm^−1^ could be attributed to the stretching vibration of the azide group. It also confirmed the successful synthesis of the previous step.

And for NCS, the peak at 2106 cm^−1^ disappeared. At the same time, absorptions at 1774 cm^−1^ and 1709 cm^−1^ also disappeared, which seemed to indicate that the azide group and *N*-phthaloyl groups were removed. Meanwhile, the peak at around 1600 cm^−1^ was assigned to the –NH_2_ group. Finally, for QCS and DQCS, the peak at 1473 and 1477 cm^−1^ was ascribed to the characteristic absorption of N–CH_3_, respectively [22]. Finally, the quaternization intensity of DQCS was stronger than that of QCS.

#### 2.1.3. NMR Analysis

**Figure 2:**
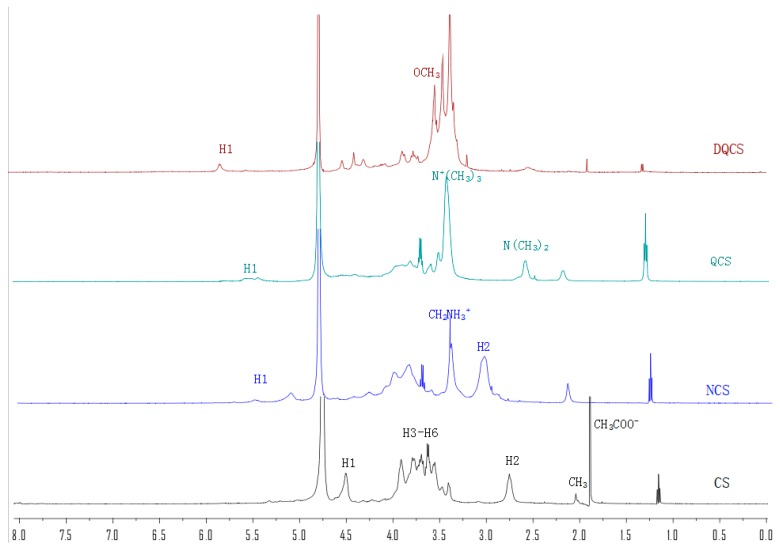
^1^H NMR spectra of chitosan derivatives.

The ^1^H NMR spectra of chitosan derivatives are showed in Figure 2. In the ^1^H NMR spectrum of CS, the peaks at 2.1 ppm and 1.9 ppm corresponded to the CH_3_ group and CH_3_COO^−^ group, respectively. The peak at around 2.8 ppm was attributed to [H2], the peaks at 3.9 ppm to 3.4 ppm were attributed to [H3]–[H6], and the peak at 4.5 ppm was attributed to [H1]. The signal at 4.8 ppm was related to the solvent D_2_O, and the signals at 3.6 ppm and 1.2 ppm were related to th eresidue of ethanol. The ^1^H NMR spectra of NCS, QCS, and DQCS showed a multiplet signal at 5.8 ppm to 5.1 ppm, corresponding to the C-1 of the glycopyranose unit [H1], and the signals from 4.5 ppm to 3.7 ppm were attributed to the non-anomeric protons [H3, H4, H5, and H6]. For NCS, the new peak at 3.3 ppm was attributed to –CH_2_NH_3_^+^ at C-6, which suggested that the amino groups were incorporated into the C-6 position of chitosan [23]. At the same time, [H2] was shifted to a lower field (3.1 ppm) compared with the signal of CS when D_2_O/DCl was used as a solvent [24,25]. For QCS, the signal at 3.3 ppm was attributed to N^+^(CH_3_)_3_ and the weaker signal at 2.5 ppm was attributed to N(CH_3_)_2_ at C-2. In additional, the peak at 3.5 ppm should be the OCH_3_ peak.

*O*-Methylation% = [[O(CH_3_)]/[H-2, H-3,H-4, H-5, H-6, H-6′] × 6/6] × 100 = 23%.

For DQCS, the double peak at 3.4 ppm and 3.3 ppm was attributed to the hydrogen groups of the methyl units substituted at C-2 and C-6. Similarly, the *O*-methyl peak could be seen in the spectra.

*O*-Methylation% = [[O(CH3)]/[H-2, H-3,H-4, H-5, H-6, H-6′] × 6/6] × 100 = 38%.

^1^H NMR and ^13^C NMR chemical shifts of chitosan derivatives are shown in Tables S1 and S2.

According to the results, there were no monomethyl and very weak dimethyl groups signals in the ^1^H NMR spectrum, indicating that we obtained a highly quaternized product. The degree of quaternization (DQ) was determined from the ^1^H NMR spectra as represented in the following equation:

(1) $$\mathrm{DQ}\left(\%\right)=\left[\frac{-N^{+}\left({\mathrm{CH}}_{3}\right){\;}_{3}\;}{9\times \left[H1\right]}\right]\times 100$$

where DQ(%) is the degree of quaternization, N^+^(CH_3_)_3_ is the integral area under the peak of the *N*,*N*,*N*-trimethyl hydrogen atom at 3.3–3.4 ppm, and [H1] is the integral area under the peak of the H1 hydrogen atom at 5.9–5.2 ppm [26]. The DQ of QCS and DQCS was determined as 0.8 and 1.3, respectively (Figures S1 and S2). These data proved the synthesis of QCS and DQCS.

#### 2.1.4. Thermogravimetric Analysis (TGA)

**Figure 3:**
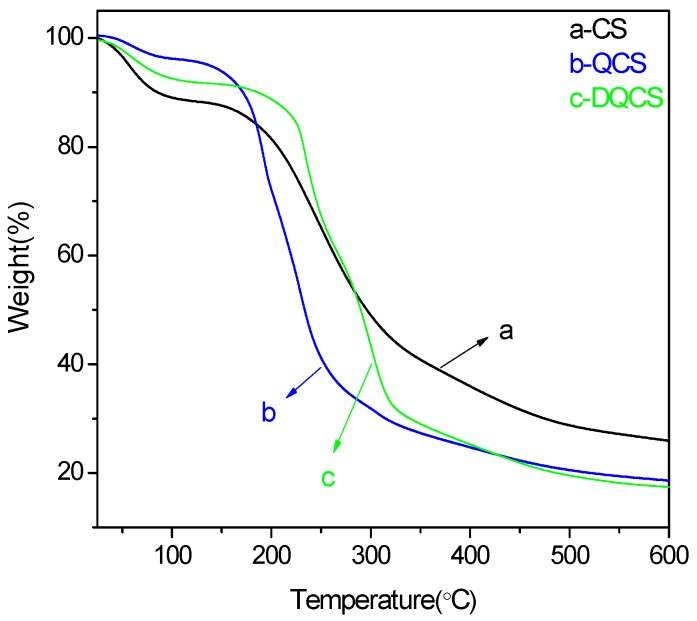
TGA thermogram curves. (**a**) CS; (**b**) QCS; (**c**) DQCS.

Thermal gravimetric analysis was used to investigate the thermal degradation and crystallization of the polymers. Figure 3 shows the TGA curves of CS, QCS, and DQCS. The CS shows two major peaks on the TG curves. CS underwent a 11.6% loss of mass between 30 and 122 °C, which resulted from the evaporation of water already within the polymer structure [27]. In the second step, between 140 and 386 °C, 50.8% mass loss was observed. This mass loss could be attributed to the degradation of the polysaccharide structure of the molecule [1]. A comparatively similar behavior of TGA was observed, as seen in QCS and DQCS. QCS showed a 4.3% loss of mass between 41 and 116 °C during the first step. In the second step, between 120 and 323 °C, 63.5% mass loss was observed. This mass should be due to the breaking of intermolecular hydrogen bonding and the glycoside linkages [28]. DQCS showed a 7.9% loss of mass between 41 and 138 °C during the first step. In the second step, between 140 and 390 °C, 67.4% mass loss was observed.

In this study, the weight loss of CS, QCS, and DQCS was 73.9%, 81.8%, and 82.1%, respectively. This poor thermal stability of chitosan derivatives suggested that the groups of quaternized salts resulted in the breakage of the hydrogen bond of CS. It was found that increasing the number of substituted groups would reduce the thermal stability of the sample, which is consistent with previous reports [9].

#### 2.1.5. Scanning Electron Microscopy (SEM) Analysis

**Figure 4:**
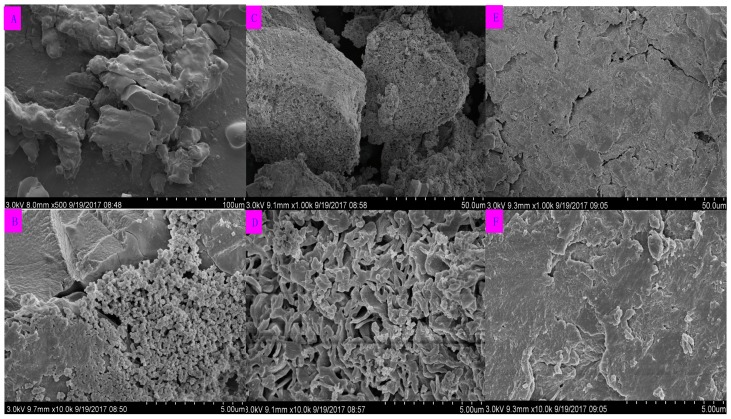
Surface morphology of CS (**A**,**B**), QCS (**C**,**D**), DQCS (**E**,**F**).

The morphology of CS, QCS, and DQCS were studied by SEM (Figure 4), by studying images with different magnifications at different areas of samples. The SEM images of CS exhibited a smooth surface, but with spherical particles in some areas. QCS shwed a homogeneous porous structure and DQCS had a compact and flat morphology. It was found that quaternization products differ in size and shape. According to the results, we conclude that quaternization results in an obvious change in the surface morphology of the prepared materials. It was concluded that the loose and porous structure of the QCS resulted in thermal properties that were not as stable as those of CS and DQCS. This was in accordance with the TGA/DTG results showing that the temperature at which the maximum degradation of QCS, DQCS, and CS occurred was 192 °C, 234 °C, and 246 °C, respectively.

### 2.2. Antioxidant Activities

#### 2.2.1. DPPH-Radical Scavenging Ability Assay

**Figure 5:**
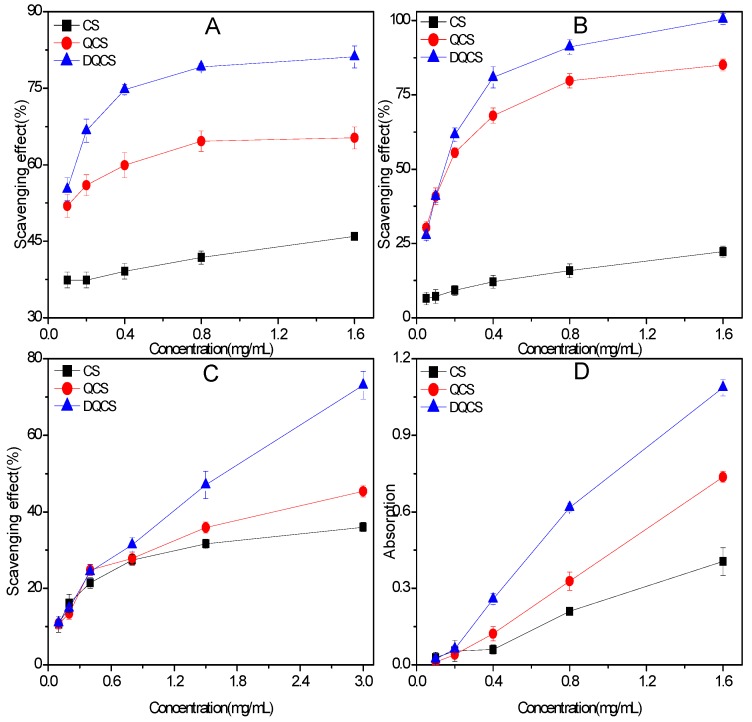
Antioxidant effect of samples: (**A**) DPPH-radical scavenging ability; (**B**) Hydroxyl-radical scavenging ability; (**C**) Superoxide-radical scavenging ability; (**D**) Reducing power.

DPPH is a useful reagent for investigating the free radical-scavenging activity of compounds. This method is based on the reduction of alcoholic DPPH solution in the presence of an antioxidant into non-radical DPPH-H, and the reduction in color is monitored over time [29]. The scavenging property of CS, QCS, and DQCS against DPPH-radical at various concentrations is showed in Figure 5A. The scavenging effect increased as the concentration of the polymer samples increased to a certain extent, and then levelled off with an even further increase in the concentration. The scavenging ability against DPPH-radical was in the order of DQCS > QCS > CS in the tested concentration range. The results showed that DQCS and QCS exhibited remarkable improvement on DPPH-radical scavenging activity; the scavenging effect were 81.2% and 65.3% at 1.6 mg/mL, respectively. By contrast, the scavenging effect of CS was only 37.4%. Meanwhile, the 50% inhibition concentration (IC_50_) values of samples were also calculated to assess their antioxidant property. A lower IC_50_ value indicates greater antioxidant activity (Table 1). This result showed that both DQCS and QCS had the highest activity upon the elimination of DPPH-radical compared to CS. The IC_50_ of DQCS and QCS was 0.05 mg/mL.

The scavenging activity may be related to the reaction of DPPH-radical with active hydrogen in chitosan derivatives to form a more stable macromolecule radical. It is reported that the active hydroxyl in the chitosan backbone plays a more important role in DPPH• scavenging than the amino [30]. Moreover, it is well known that chitosan has strong intra- and intermolecular hydrogen bonds; as a result, OH is difficult to dissociate. Compared with CS, DQCS and QCS have strong electron-withdrawing methyl groups. The electron-withdrawing groups improve the energy level of the highest occupied molecular orbital (HOMO) and decline the dissociation energy of O–H [31]. The more electron-withdrawing groups, the more active hydrogen. Therefore, DQCS exhibited the highest DPPH• scavenging ability, and the positive charge on the nitrogen atoms (introduced by methyl groups) might also increase DPPH• scavenging activity. In other words, quaternized groups are an important factor that influences the scavenging activity against DPPH radical.

#### 2.2.2. Hydroxyl-Radical Scavenging Ability Assay

Hydroxyl radical is the most reactive free radical and can be formed from a superoxide anion and hydrogen peroxide, which can react with living cells and induce severe damage [32]. Figure 5B showed the curve chart of the hydroxyl radical scavenging ability of CS, QCS, and DQCS at various concentrations. The results were similar to the results of the DPPH radical scavenging activity. According to the graph, we conclude the results as follows. Firstly, the scavenging indices enhanced with increasing concentration. Secondly, the scavenging ability against hydroxyl radicals was in the order of DQCS > QCS > CS in the tested concentration range. Thirdly, DQCS, QCS, and CS showed antioxidant activity of 100%, 85.1%, and 22.2% at 1.6 mg/mL, respectively. In addition, as shown in Table 1, the scavenging activity of DQCS on hydroxyl radical was higher than that of QCS. The IC_50_ of DQCS and QCS was 0.14 mg/mL and 0.16 mg/mL, respectively.

Our results were mainly affected by two factors: one is that the active O–H in the polysaccharide unit can react with •OH by the typical H abstraction reaction, and this principle has been noted to influence the DPPH-radical scavenging assay. The other is that the positive charge could attract the single electron of free radicals to damage the free radical chain reaction [15]. Nitrogen atoms bonded to the methyl groups can be protonated and, consequently, the positive charge density on QCS and DQCS is strengthened in water. Thus, DQCS had the highest scavenging ability. We conclude that quaternized groups could be a positive factor that affects the scavenging activity against hydroxyl radical.

#### 2.2.3. Superoxide-Radical Scavenging Ability Assay

Superoxide anion is one of the precursors of singlet oxygen and hydroxyl radicals, which can damage cells and DNA, leading to various diseases [33]. Superoxide scavenging activity was determined by an NBT assay. As shown in Figure 5C, the superoxide-radical scavenging ability of the obtained derivatives was similar to the scavenging properties against hydroxyl radicals and DPPH radicals. Also, the scavenging ability of polymer samples increased with the concentration. It was DQCS that had the best enhanced scavenging activity, reaching 73.1% at 3.0 mg/mL. The maximum of 45.4% and 36% inhibition was observed at the concentration of 3.0 mg/mL for QCS and CS. Moreover, as given in Table 1, the scavenging activity of DQCS on superoxide radical was higher than that of QCS. The IC_50_ of DQCS and QCS was 1.7 mg/mL and 3.7 mg/mL, respectively.

As is known, the scavenging effect is related to the number of active hydroxyl groups in the molecule. As determined by the DPPH radical and hydroxyl radical assays, the quaternary ammonium salts grafted on CS can donate active hydrogen and the positive charge that reacts with superoxide anion to form a more stable macromolecule radical. Therefore, more quaternized groups and a higher positive charge density in DQCS promotes the scavenging effect. The results again suggest that DQCS can be considered as efficient antioxidant polymer, and that quaternized groups play a role in free radical scavenging ability.

#### 2.2.4. Reducing Power Assay

The ferric ion reducing power assay tests reducing power based on an electron transfer reaction. In the assay, the presence of reductants (antioxidants) results in the reduction of the ferric ion/ferricyanide complex to the ferrous form, with a characteristic formation of Perl’s Prussian blue, which can be measured spectrophotometrically [34]. From Figure 5D, we can clearly see that the reducing power of polymer samples correlated well with the increasing concentration. DQCS exhibited a stronger reducing power than QCS and CS, and they showed a reducing power of 1.1, 0.7, and 0.4 at 1.6 mg/mL, respectively.

Accordingly, the reducing capacity of a compound may serve as a significant indicator of its potential antioxidant activity [35]. Our results on the reducing power suggest that quaternary ammonium salts likely contribute significantly toward the reducing power.

## 3. Experiment

### 3.1. Materials

Chitosan of low molecular weight was supplied by Zhejiang Golden-Shell Pharmaceutical Co. Ltd. (Yuhuan, China) with a deacetylation degree of 90%. The deacetylation degree (DD) was determined by elemental analysis. The other reagents, such as *N*,*N*-Dimethylformamide (DMF), *N*-bromobutanimide (NBS), triphenylphosphine (Ph_3_P), *N*-Methyl pyrrolidone (NMP), methyl iodide (CH_3_I), sodium iodide (NaI), 1,1-diphenyl-2-picrylhydrazyl (DPPH), etc., were supplied by Sinopharm Chemical Reagent Co., Ltd., Shanghai, China.

### 3.2. Analytical Methods

FTIR spectra were measured on a Jasco-4100 Fourier Transform Infrared Spectrometer (JASCO Co., Ltd., Shanghai, China) with KBr disks. The elemental analyses (C, H, and N) were performed on a Vario Micro Elemental Analyzer (Elementar, Langenselbold, Germany). The UV–Vis absorbance was measured with a T6 New Century UV spectrometer (P General Co., Ltd., Beijing, China). 13C Nuclear Magnetic Resonance (13C NMR) spectra were measured with a Bruker AVANCE III Spectroscope (Bruker Tech. and Serv. Co., Ltd., Beijing, China). The degrees of substitution (DS) were calculated on the basis of the percentages of carbon and nitrogen. The thermogravimetric analysis (TGA) was performed using a TGA/DSC1/1100 (Mettler-Toledo). The X-ray diffraction patterns of samples were recorded at room temperature on an X-ray diffractometer (D8 advance, Bruker, Germany). The morphology of the samples was examined through a scanning electron microscope (SEM) (S-4800, Hitachi, Tokyo, Japan). Each sample was coated with gold in an ion sputter (E-1045, Hitachi, Japan) before being scanned and photographed at specific magnifications (1000×). An accelerating potential of 3 kV was used during image acquisition.

### 3.3. Synthesis

#### 3.3.1. 6-Deoxy-*N*-phthaloyl-chitosan (**2**)

First, 15.1 g of phthalic anhydride was dissolved in 300 mL DMF containing 5% (*v*/*v*) water. Then, 5.1 g of CS was added to the solution, and the mixture was heated at 120 °C. After 9.5 h of reaction, the resulting mixture was cooled to room temperature and gradually poured into ice water. The precipitate was collected on a filter, washed twice with distilled water and ethanol, and dried at 60 °C. The product was obtained as a flesh-colored powdery material. Yield: 57%.

#### 3.3.2. Preparation of 6-Bromo-6-deoxy-*N*-phthaloyl-chitosan (**3**)

First, 3.5 g phthaloylated chitosan (2) was dissolved in 330 mL DMF, then 21.4 g NBS and 31.4 g triphenylphosphine were added slowly in an ice bath. The mixture was stirred at 80 °C for 2 h. The reaction mixture was poured into ethanol, and the resulting precipitate was collected by filtration. Yield: 40%.

#### 3.3.3. Preparation of 6-Azido-6-deoxy-*N*-phthaloyl-chitosan (**4**)

A mixture of 1.6 g 6-bromo-6-deoxy-*N*-phthaloyl-chitosan (2) and 50 mL DMF was treated with sodium azide (2.0 g) at 100 °C for 7 h. The resulting mixture was cooled to room temperature and stirred overnight. The solution was then poured into 400 mL ice water. The precipitate was collected, washed with distilled water and ethanol, and then dried at 60 °C to give the product. Yield: 86%.

#### 3.3.4. Preparation of 6-Amino-6-deoxy chitosan (NCS)

First, 0.95 g 6-azido-6-deoxy-*N*-phthaloyl-chitosan (**4**) was suspended in 100 mL DMF. Then 2.67 g triphenylphosphine was added. After 20 h of reaction at room temperature, 95 mL hydrazine monohydrate was then added to the mixture and the mixture was heated to 100 °C. After 8 h of reaction, the resulting solid was washed with 200 mL ethanol and dried to give 6-amino-6-deoxychitosan. Yield: 58%.

#### 3.3.5. Preparation of DQCS

A mixture of NCS (0.48 g, 3 mmol), 1.05 g sodium iodide, 3 mL aqueous sodium hydroxide solution (15%, *w*/*v*), and 3 mL iodomethane in 10 mL NMP stirred at 60 °C for 2 h. The mixture was precipitated into ethanol, and the precipitate was collected by filtration and washed by ethanol. The products were dried at 60 °C for 24 h. Moreover, we carried out the reaction three times with the same quantity of iodomethane. Yield: 61%.

#### 3.3.6. Preparation of QCS

The synthesis method of QCS is similar to that of DQCS, except that the amount of aqueous sodium hydroxide solution and iodomethane was halved.

After obtaining the quaternary ammonium salt, we performed a two-day dialysis with pure water. We changed the water eight to 10 times during this period. That is, the samples were dissolved in pure water (about 50 mL) and then dialyzed with pure water (about 800 mL). Then we changed the water every 3 h (except night). As a result, the content of iodine ion was expected to be low. We speculate that iodide has little effect on the antioxidant effect.

### 3.4. Investigation of the Antioxidant Ability

#### 3.4.1. DPPH-Radical Scavenging Ability Assay

The DPPH scavenging properties of the products were evaluated by the following method [36]. Different concentrations of samples (CS, QCS, and DQCS) and 2 mL DPPH ethanol solution (180 μmol/L) was incubated for 30 min at room temperature. Then, the absorbance of the remaining DPPH radical was measured at 517 nm against a blank. Three replicates for each sample were tested and the scavenging effect was calculated according to the following equation:

(2) $$\mathrm{Scavenging}\;\mathrm{effect}\;\left(\%\right)=\left[1-\frac{A_{\mathrm{sample}\;517\;\mathrm{nm}}\;-\;A_{\mathrm{control}\;517\;\mathrm{nm}}\;}{A_{\mathrm{blank}\;517\;\mathrm{nm}}}\right]\times 100$$

where *A*_sample517nm_ is the absorbance of the sample (with DPPH) at 517 nm; *A*_control517nm_ is the absorbance of the control (without DPPH) at 517 nm; and *A*_blank517nm_ is the absorbance of the blank (without samples) at 517 nm.

#### 3.4.2. Hydroxyl-Radical Scavenging Ability Assay

The test of the hydroxyl-radical scavenging ability was carried out according to Hu’s methods [37]. The reaction mixture, with a total volume 4.5 mL containing testing samples (CS, QCS, and DQCS), was incubated with EDTA-Fe^2+^ (220 μmol/L), safranine T (0.23 μmol/L), and H_2_O_2_ (60 μmol/L) in potassium phosphate buffer (150 mM, pH 7.4) for 30 min at 37 °C. The absorbance of the mixture was measured at 520 nm. Three replicates for each sample were tested and the scavenging effect was calculated according to the following equation:

(3) $$\mathrm{Scavenging}\;\mathrm{effect}\;\left(\%\right)=\left[\frac{A_{\mathrm{sample}\;520\;\mathrm{nm}}\;-\;A_{\mathrm{blank}\;520\;\mathrm{nm}}\;}{A_{\mathrm{control}\;520\;\mathrm{nm}}-A_{\mathrm{blank}\;520\;\mathrm{nm}}}\right]\times 100$$

where *A*_sample520nm_ is the absorbance of the sample at 520 nm; *A*_control520nm_ is the absorbance of the control (distilled water instead of H_2_O_2_) at 520 nm; and *A*_blank520nm_ is the absorbance of the blank (distilled water instead samples) at 520 nm.

#### 3.4.3. Superoxide-Radical Scavenging Ability Assay

The superoxide-radical scavenging ability was assessed following the model of Wei’s methods with minor modifications [12]. Involving testing samples (CS, QCS, and DQCS), 30 μmol phenazine mothosulfate (PMS), 338 μmol nicotinamide adenine dinucleotide reduced (NADH), and 72 μmol nitro blue tetrazolium (NBT) in Tris–HCl buffer (16 mM, pH 8.0), the reaction mixture was incubated at 25 °C for 5 min. The absorbance of the mixture was measured at 560 nm. Three replicates for each sample were tested and the scavenging effect was calculated according to the following equation:

(4) $$\mathrm{Scavenging}\;\mathrm{effect}\;\left(\%\right)=\left[1-\frac{A_{\mathrm{sample}\;560\;\mathrm{nm}}\;-\;A_{\mathrm{control}\;560\;\mathrm{nm}}\;}{A_{\mathrm{blank}\;560\;\mathrm{nm}}}\right]\times 100$$

where *A*_sample560nm_ is the absorbance of the sample at 560 nm; *A*_control560nm_ is the absorbance of the control (distilled water instead of NADH) at 560 nm; and *A*_blank560nm_ is the absorbance of the blank (distilled water instead samples) at 560 nm.

#### 3.4.4. Reducing Power Assay

The reducing power was determined according to the method of Zhong [38]. Briefly, 1.5 mL testing sample was mixed with 1.5 mL 1% potassium ferricyanide, and the mixture was incubated at 50 °C for 20 min. Thereafter, 1.5 mL 10% trichloroacetic acid was added. The upper layer (2.0 mL) was mixed with 2.0 mL water and 0.2 mL 0.1% ferric chloride, and the absorbance was measured at 700 nm. A higher absorbance indicates a stronger reducing power [39].

## 4. Conclusions

In summary, a series of derivatives of chitosan with single or double quaternary ammonium salts were synthesized successfully. In addition, the antioxidant abilities of chitosan and quaternized chitosan derivatives were tested in vitro. In our case, the antioxidant ability of chitosan derivatives was improved in all samples tested. The mechanism for their antioxidant abilities was also discussed in this paper. In short, quaternized groups could weaken the dissociation energy of the O–H band that causes polysaccharide to donate more active hydrogen and increase the positive charge density as well. On the basis of the above results, it could be concluded that the antioxidant abilities are influenced by quaternized groups. Thus, DQCS, with more quaternized groups and higher positive charge density, would have stronger antioxidant abilities. Collectively, these studies add to the body of evidence that DQCS has antioxidant capacities and can be used as a promising candidate material in pharmaceutical and food industries.

## Figures and Tables

**Table 1:** Antioxidant activity of chitosan derivatives.

Radical	CS (mg/mL)	QCS (mg/mL)	DQCS (mg/mL)
DPPH (IC_50_)	2.4	0.05	0.05
Hydroxyl (IC_50_)	7.7	3.7	1.7
Superoxide (IC_50_)	5.1	0.16	0.14

## Supplementary Materials

The supplementary materials are available online.

## Abbreviations

The following abbreviations are used in this manuscript:

FTIR	Fourier transform infrared
^1^H-NMR	^1^H nuclear magnetic resonance
TGA	Thermogravimetric analyses
SEM	Scanning electron microscopy
NMP	*N*-methyl-2-pyrrolidone
DMF	*N*,*N*-Dimethylformamide
DPPH	1,1-Diphenyl-2-picrylhydrazyl
PMS	phenazine mothosulfate
NADH	nicotinamide adenine dinucleotide
NBT	nitro blue tetrazolium
