# Supplementary material for: Preparation and Characterization of Quaternized Chitosan Derivatives and Assessment of Their Antioxidant Activity
Source: Molecules. 2018 Feb 26;23(3):516. doi: 10.3390/molecules23030516 (PMC6017865; doi:10.3390/molecules23030516)
Supplement: Supplementary file 1 [file molecules-23-00516-s001.pdf]

Preparation and Characterization of Quaternized Chitosan Derivatives and Assessment of Their Antioxidant Activity

Fang Luan [1,2], Lijie Wei [1,2], Jingjing Zhang [1,2], Wenqiang Tan [1,2], Yuan Chen [1,2], Fang Dong [1], Qing Li [1], Zhanyong Guo [1,2,*]

[1] Key Laboratory of Coastal Biology and Bioresource Utilization, Yantai Institute of Coastal Zone Research, Chinese Academy of Sciences, Yantai 264003, Shangdong, China; fluan@yic.ac.cn (F.L.); ljwei@yic.ac.cn (L.W.); jingjingzhang@yic.ac.cn (J.Z.); wqtan@yic.ac.cn (W.T.); fdong@yic.ac.cn (F.D.); qli@yic.ac.cn (Q.L.)
[2] University of Chinese Academy of Sciences, Beijing 100049, China
* Correspondence: zhanyongguo@hotmail.com (Z.G.);

Tel.: +86-535-2109171 (Z.G.); fax: +86-535-2109000 (Z.G.)

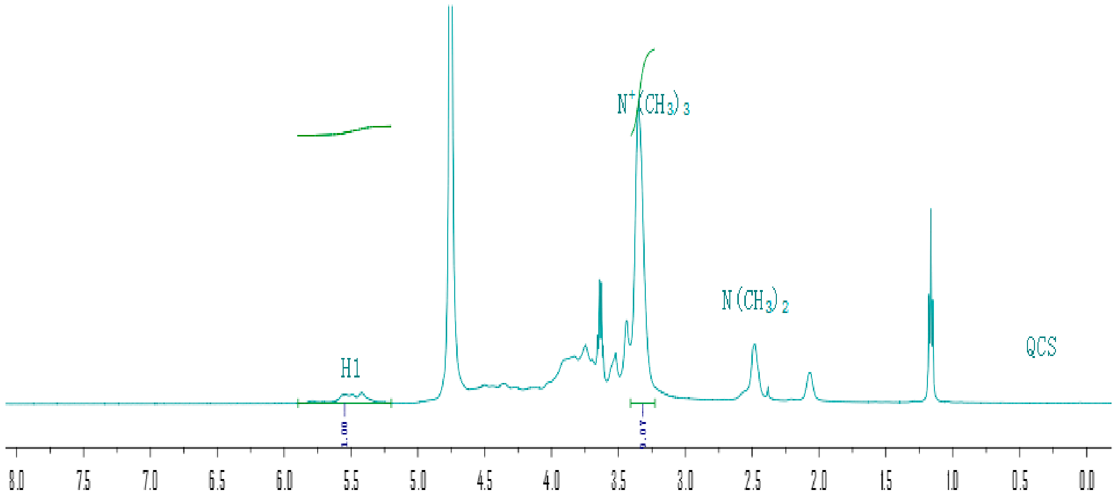

Fig. S1. ^{1}H NMR spectra of QCS in D_2O.

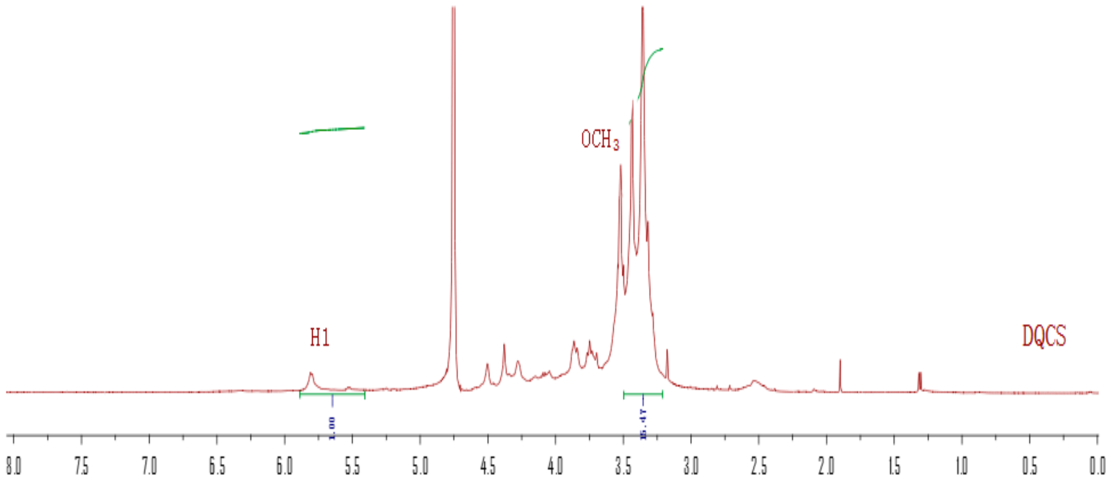

Fig. S2. ^{1}H NMR spectra of DQCS in D_2O.

Table S1. ^{1}H NMR chemical shifts of chitosan derivatives.

CS	4.5 ppm (H1)	2.8 ppm (H2)		3.4-4.0 ppm (H3-H6)
NCS	5.1-5.5 ppm (H1)	3.1 ppm (H2)		3.4-4.3 ppm (H3-H6)
QCS	5.3-5.7 ppm (H1)	3.5-4.3 ppm (H2-H6)	3.3 ppm ([N(CH_3)$_3$])	3.4-3.5 ppm (OCH_3)
DQCS	5.5-5.9 ppm (H1)	3.6-4.6 ppm (H2-H6)	3.3-3.4 ppm ([N(CH_3)$_3$])	3.5 ppm (OCH_3)

Table S2. ^{13}C NMR chemical shifts of chitosan derivatives.

sample	Peak (ppm)
CS	104.9 (C1), 78.0 (C4), 75.0 (C5), 69.8 (C3), 60.7-60.2 (C6), 57.4 (C2)
QCS	98.0 (C1), 77.5-76.9 (C2,OCH_3), 74.7 (C4), 73.0 (C5), 58.6-58.3 (C6), 54.1 [(NCH_3)$_3$], 50.1 [(NCH_3)$_2$]
NCS	105.4 (C1), 82.2-75.7 (C3,C4,C5), 57.6 (C2), 40.4 (C6)
DQCS	97.8 (C1), 77.4-76.7 (C2,OCH_3), 74.7 (C4), 72.9(C5), 58.3-58.1 (C6), 55.4-54.0 [(NCH_3)$_3$]
